# Tribomechanical Properties of Glazes for Ceramic Tiles: A Novel Protocol for Their Characterization

**DOI:** 10.3390/ma18010060

**Published:** 2024-12-27

**Authors:** Riccardo Fabris, Giulia Masi, Denia Mazzini, Leonardo Sanseverino, Maria Chiara Bignozzi

**Affiliations:** 1 Department of Civil, Chemical, Environmental and Materials Engineering (DICAM), University of Bologna, Via Terracini 28, 40131 Bologna, Italy; riccardo.fabris2@unibo.it (R.F.); giulia.masi5@unibo.it (G.M.); 2 Colorobbia Italia S.p.A., Via Bucciardi 37, Fiorano Modenese, 41042 Modena, Italy; mazzinid@colorobbia.it; 3 Centro Ceramico, Via Valle d’Aosta 1, Sassuolo, 41049 Modena, Italy; sanseverino@centroceramico.it

**Keywords:** tile, glass-ceramic, glaze, surface, wear resistance

## Abstract

The aim of the work is to design and validate a characterization protocol for glazes used in the ceramic tile industry to lead manufacturers and researchers towards the formulation of glazes with enhanced wear resistance properties. The focus of the protocol is addressed to determine surface parameters that strongly depend on glaze formulation and firing temperature. This protocol includes analytical (e.g., thermal analysis, Vickers microhardness, microstructural investigation, etc.) and technological tests (i.e., impact resistance and surface abrasion resistance test), the latter carried out on ceramic tile samples where four different glazes have been applied. The characterization protocol set in this paper highlights the importance of using both analytical and technological tests for glaze investigations and provides threshold values for specific parameters useful in developing glass-ceramic glazes with enhanced mechanical and tribological properties.

## 1. Introduction

Over the past two decades, the ceramic tile manufacturing industry has seen significant technological progress to address the expectations of European and international markets. This progress has allowed the development of manufacturing techniques, enabling the production of sustainable and high-quality ceramic tiles with a reduced environmental impact [1,2,3,4,5]. Furthermore, the demand for ceramic tiles with aesthetic features associated with enhanced tribomechanical performances and high durability is becoming imperative in view of their different applications (e.g., floorings, walls, kitchen countertops, etc.) [5,6,7,8,9,10].

The top layer of ceramic tile is usually made of glaze, and its role is fundamental to prevent scratches, wear, and impact damages and to increase surface durability, thus extending the ceramic tile lifespan [10,11,12,13]. In the past, glazes for ceramic tiles were primarily amorphous and formulated with raw materials and frits unable to crystallize during firing [8]. Although these glazes exhibited excellent transparency, thus preserving the decoration, they were characterized by limited mechanical and durability performances [5,8,9]. To overcome these limits, extensive research has been carried out to design new glass-ceramic glazes (GCGs) featuring enhanced tribomechanical performance [10,11,12,14,15,16,17]. GCGs are typically defined as polycrystalline materials in which crystalline structures are embedded in an amorphous matrix [18] and their mechanical and tribological properties have been extensively investigated using analytical techniques such as Vickers microhardness, thermal analysis, microstructural and morphological investigations, and optical measurements [11,12,13,14,19,20,21]. Although numerous scientific studies in the literature assess the performance of GCGs using the previously mentioned tests, a different approach is adopted in the glaze industry, where characterization tests are mainly performed following EN and/or ISO standards. In particular, ISO 13006 and EN 14411 [22,23] play a crucial role for ceramic tiles, as they establish classification criteria and the requirements for the main characteristics, including surface properties such as stain and abrasion resistance that are strictly related to glaze properties. The physical, chemical, and mechanical properties of ceramic tiles are verified by means of test methods reported in ISO 10545 parts 1–25. However, only a few scientific publications are available in the literature where test methods according to EN/ISO standards are adopted for surface characterization [10,11,12,13,24,25]. Indeed, these methods, which can be defined as “technological tests”, are not sufficient to provide results useful for a full comprehension of various phenomena occurring during glaze formulation and sintering.

This paper aims to develop and validate a glaze characterization protocol (GCP) that combines analytical and technological tests useful for glaze manufacturers and researchers involved in new glaze formulations. The focus of the protocol is to determine surface parameters that strongly depend on glaze formulation and firing temperature, thus leading to a fine-tuned characterization able to quickly direct the glaze formulations towards the desired features, such as improved tribological properties.

Four commercial glazes with various compositions have been used for GCP validation, and target values for Vickers hardness, gloss variation, and scratch resistance have been identified as main indicators for the development of further tribological properties. The combination of specific analytical test methods with technological ones has been found to be the key for the development of glaze formulations able to satisfy the needs of the ceramic tiles market, which is getting more and more demanding.

## 2. Glaze Characterization Protocol (GCP) Design

**Figure 1:**
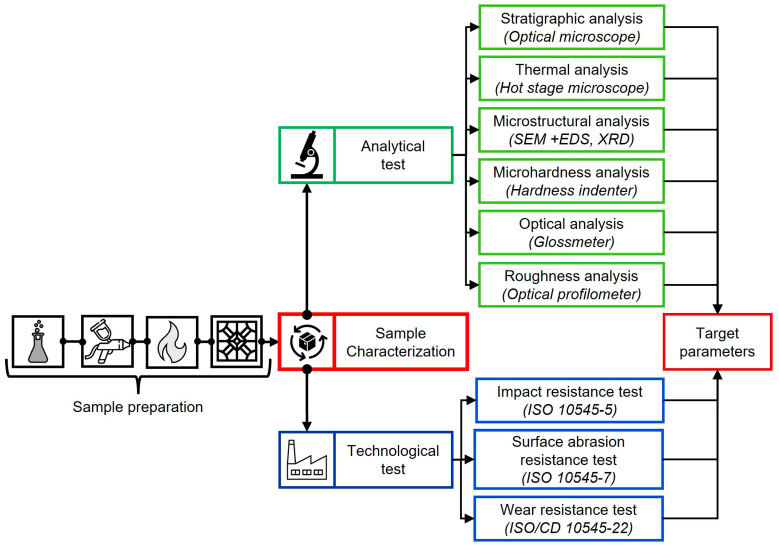
Schematic representation of GCP.

The glaze characterization protocol (Figure 1) has been developed primarily by considering properties that shall be determined on the glaze layer, which is jointly fired with the ceramic tile body. The first steps of the protocol address sample preparation, closely replicating the industrial manufacturing process, which involves the airless application of engobe and glaze coatings to the ceramic tile body, followed by drying and firing steps.

The selection of the properties and relevant tests to be included in the protocol has been performed based on literature research [9,11,14,16,26,27,28,29,30,31,32,33,34] and on technical standards commonly used in the ceramic tile sector. In particular, the investigation of the following properties by analytical tests was considered fundamental: (i) the glaze thermal properties, such as their sintering, softening, and sphere temperatures, using a hot stage microscope; (ii) glaze microstructural characteristics, such as size and distribution of closed porosities, by optical microscope as well as shape, size, distribution, chemical composition, and nature of crystalline structures by scanning electron microscopy and X-rays diffraction analysis; and (iii) surface parameters such as Vickers microhardness, surface reflectivity, and roughness parameters by Vickers indenter, glossmeter, and optical profilometer, respectively. All these properties need to be determined and/or monitored during new glaze formulations process if high tribological characteristics are desired.

As far as technological properties are concerned, the investigations were carried out using some ceramic tile test methods reported in ISO 10545 parts 1 to 25. Among all the available standards, those addressing surface characteristics, such as impact (ISO 10545-5 [35], surface abrasion ISO 10545-7 [36], and wear resistance, were selected. Wear resistance has been determined by the test method described in ISO/CD 10545-22 [37], which is currently under discussion in the framework of WG1 “Test methods” of ISO TC 189 “Ceramic tiles” as previously reported [38]. This novel method allows for the determination of ceramic tile wear resistance by exploiting a combination of different test methods such as scratch resistance, gloss variation, and weight loss after surface abrasion. Additionally, the method provides a classification based on the results of each test to identify the most suitable intended use for the tested ceramic tiles.

The choice to select ISO standards was based on the following issues: (i) international standards are known and applied worldwide; (ii) ceramic tile production is well established worldwide, and the relevant market is global with imports/exports all over the world; (iii) standards such as CSTB Cahier 3778 Annex 10 (France) [39] and ASTM C1870-18 (USA) [40] for determining impact and abrasion resistance, respectively, are mainly applied only in the USA and France.

## 3. Materials and Methods

### 3.1. Glazes and Samples Preparation

Four different glaze formulations for porcelain stoneware floor tiles were considered for the validation of the glaze characterization protocol. Glaze and ceramic tile body preparation was carried out at Colorobbia Italia S.p.A (Fiorano Modenese, Modena, Italy). The four glazes (named G1, G2, G3, and G4) were produced by mixing different types of commercially available frits (named F1, F2, F3, and F4) and raw materials, according to Table 1. The chemical compositions of all the considered frits and raw materials are reported in Table 2.

The chemical composition of the investigated glazes is reported in Table 3, highlighting that G1, G2, and G4 were designed within the framework of the NaO-K_2_O-ZnO-CaO-MgO-SiO_2_-Al_2_O_3_-BaO system, whereas G3 was designed within the framework of NaO-K_2_O-ZnO-CaO-MgO-SiO_2_-Al_2_O_3_-B_2_O_3_. The chemical composition of frits and glazes has been determined through an energy-dispersive X-ray fluorescence spectrometer (EDXRF, PANalytical Epsilon 4) equipped with an X-ray tube with an Ag anode, operating at a voltage of 10 kV and a current intensity of 2 mA. Measurements were performed on powdered samples prior to firing, which were milled and sieved to a particle size smaller than 0.063 mm.

Each glaze was prepared as follows: 500 g of the mix combined with 0.1 wt% sodium tripolyphosphate and 0.3 wt% carboxymethyl cellulose were wet-milled for 45 min in a ceramic jar with 40 wt% water and 500 g of alumina balls. The milled mixture was sieved through a 200-mesh sieve to remove any coarser particles, and water was added, when necessary, to reach a density of 1450 kg/m^3^, suitable for airless application.

Each glaze was applied on three 30 × 30 cm unfired stoneware tile bodies, previously coated by a white engobe having a density of 1470 kg/m^3^ to achieve a uniform color over the tile body surface. The airless application of both the white engobe and the glaze was carried out in the amount of 44 mg/cm^2^. All the coated samples were dried in a ventilated electric oven at 110 °C for 60 min and then air-cooled. Subsequently, the dried samples were single-fired in an industrial roller kiln using a fast-firing cycle with a heating rate of 30 °C/min and a peak temperature (T_P_) of 1205 °C.

For the surface abrasion resistance test, two additional 30 × 30 cm ceramic tiles with light gray and dark blue decoration were prepared for each glaze formulation. Decorative patterns were printed onto the engobe layer using a digital printer (Digiglaze, System Ceramics S.p.A, Fiorano Modenese, Modena, Italy). The samples were then glazed and fast-fired following the previously described process.

### 3.2. Characterization Methods

Cross-section observations were performed on two polished samples of 10 × 10 mm glazed tile using a metallographic optical microscope equipped with a digital camera (OM, Leica DM-LM, D, Leica Microsystems S.r.l, Milano, Italy). Specimens were obtained by cutting from 30 × 30 cm tiles, and mirror-like polishing was carried out using a grinding machine. Micrographs were analyzed using image analysis software (LAS version 3.8, Leica Microsystems S.r.l, Milano, Italy) to determine the thickness of the different layers and the size of closed porosities.

Glaze characteristic temperatures, such as sintering, softening, and sphere temperatures (T_SI_, T_SO_, and T_SP_, respectively), were measured using a hot stage microscope (HSM, ELS-MDF, Expert System Solutions S.r.l, Modena, Italy) on glaze samples specifically prepared. After wet-milling, the obtained slurry was dried, further milled, and sieved through a sieve with a mesh size of 0.063 mm. The resulting powder was first pressed and then extruded to obtain cylindrical pellets with a height of 3 mm and a diameter of 2 mm. The hot stage microscope analysis was carried out on the cylindrical pellet by applying a linear heating ramp up to 1300 °C with a rate of 30 °C/min. The heating ramp was selected to be similar to the one used for the production of glazed samples. Glaze characteristic temperatures were extracted from images captured during the heating process by analyzing the cylindrical pellet shape variation through image analysis software (Misura version 3.32, Expert System Solutions S.r.l, Modena, Italy).

Microstructural analysis was carried out using a field emission gun—scanning electron microscope (FEG-SEM, Tescan Mira3, TESCAN GROUP a.s, Brno, Czech Republic) equipped with an energy-dispersive X-ray detector (EDS, Quantax EDS, Bruker Nano GmbH, Berlin, Germany) to analyze the shape, size, and distribution of crystalline phases. Samples were obtained by cutting from 30 × 30 cm tiles, and a thin gold layer was sputtered onto their surfaces using a sputter coater (Q150R ES, Quorum Technologies Ltd., West Sussex, UK), making samples conductive. Micrographs were recorded detecting backscattered electrons with a beam acceleration of 20 kV and a working distance of 10 mm.

Crystalline phases were determined by analyzing diffraction patterns acquired on glaze powders using an X-ray diffractometer (XRD, PANalytical Empyrean Series III, Malvern Panalytical Ltd., Malvern, UK). These samples were prepared by drying the glaze slurries to a constant mass, followed by milling and sieving to achieve a particle size smaller than 0.063 mm. Subsequently, 5 g of the resulting powder was placed into a cylindrical mold with a diameter of 25 mm and pressed at a working pressure of 20 bar by using a hydraulic press. For each glaze, a disk was obtained and fired using the industrial fast-firing cycle. The fired disks were further milled and sieved to a particle size smaller than 0.063 mm. XRD analysis was carried out on the glaze powder sample, rather than on the relevant glazed tile, thus avoiding signals due to the engobe layer. In this way, qualitative analysis was only representative of the glaze composition. The XRD spectra were recorded within the 2θ range of 10° to 60° with a step size of 0.02° (2θ) using a Cu-Kα source with a wavelength (λ) of 1.5406 Å, operating at a voltage of 40 kV and a current intensity of 30 mA. Crystalline phases were identified by comparing the recorded spectra with the ICSD reference spectra stored in the HighScore library (Malvern Panalytical Ltd., Malvern, UK).

Vickers microhardness measurements (HV) were performed using an microhardness tester (Isoscan Galileo, LFT S.p.A, Bergamo, Italy) equipped with image analysis software. Measurements were carried out on 10 × 15 mm samples cut from 30 × 30 cm ceramic tiles. Specifically, indentations were randomly performed on the polished cross-section along the glaze layer following the test method outlined in ASTM C1327-15, with a force of 9.81 N and a dwell time of 15 s [41]. For each sample, five measurements were carried out. Indentation traces were analyzed and measured using image analysis software.

Gloss was assessed on the glazed ceramic tile surfaces using a portable glossmeter (ZGM 1022, Zehntner GmbH Testing Instruments, Sissach, Switzerland). For each surface, 35 measurements were performed using a 60° detector. Prior to the measurements, the glossmeter was calibrated with a highly polished black glass standard provided by the manufacturer. ISO 2813 [42] defines gloss as the ratio of the luminous flux reflected from the analyzed specimen to that reflected by a highly polished black glass surface with a refractive index of 1.567 at a wavelength of 587.6 nm, multiplied by 100. This ratio is inherently dimensionless, as it is derived from the ratio of two luminous fluxes, both measured in lumens (lm). However, ISO 2813 expresses the gloss ratio in gloss units (GU), where 0 GU corresponds to a completely non-reflective surface, and 100 GU corresponds to the polished black glass specified in standard.

Roughness parameters were determined using an optical profilometer (Leica Dual Core Microscope 3D DCM 3D, Leica Microsystems S.r.l, Milano, Italy). For each sample, five roughness profiles, each 50 mm in length, were acquired using a confocal objective lens with a 20× magnification. Both the average roughness (Ra) and the maximum height of the roughness profile (Rz) were extracted from the acquired profiles following the methodologies specified in ISO 21920-2 [43].

The impact resistance test was carried out by dropping a 20 mm diameter steel ball from a height of 1 m onto the sample surface. This test was performed on five 7.5 × 7.5 cm specimens, cut from 30 × 30 cm ceramic tiles. Each of the samples considered were mounted on a 5 cm thick concrete block using a 1 mm layer of two-component resin, as specified by ISO 10545-5 [35]. After the impact test, the restitution coefficient (e), expressed as the ratio of the sphere speed before and after impact, was calculated. Additionally, the tested specimen surfaces were visually inspected to identify any presence of impact marks, fractures, or glaze chipping.

The surface abrasion resistance test was carried out on 10 × 10 cm specimens according to ISO 10545-7 [36]. Specimens used for this test were obtained by cutting from 30 × 30 cm ceramic tiles. The surface abrasion resistance class was determined by placing samples in a chamber illuminated by a 300 lux light source and visually inspecting the abraded area from a distance of 2 m and a height of 1.65 m. The outcomes of the test were related with the intended use reported in Annex-N of ISO 13006 [22].

Wear resistance was assessed on glazed surfaces following the procedures outlined in the novel method drafted in ISO/CD 10545-22 [37]. This method includes the following tests: the determination of specific weight loss (ΔWL_S_), the assessment of gloss variation (ΔG) combined with the stain resistance test, and the evaluation of scratch resistance.

ΔWL_S_ were carried out on five 10 × 10 cm specimens cut from the original 30 × 30 cm samples using the same equipment reported in ISO 10545-7 [36]. Before testing, all the samples were dried to a constant mass in an electric oven set at 110 °C. To calculate ΔWL_S_, initial (w_i_) and final (w_f_) weights of the tested sample were measured before and after 6000 abrasive cycles, respectively. The calculation was performed according to Equation (1), where A represents the abraded area in cm^2^:

∆WL_S_ = (w_i_ − w_f_)/A [mg/(cm^2^)] (1)

ΔG was evaluated according to Equation (2), where G_i_ and G_f_ represent the initial and final gloss values, respectively, after 600 or 6000 abrasion cycles, carried out with the same equipment reported in ISO 10545-7 standard. This test was performed on five 10 × 10 cm specimens, cut from the 30 × 30 cm ceramic tiles.

∆G = G_i_ − G_f_ [GU] (2)

The abraded area of each specimen was also stained by green chromium solutions prepared by adding 40 wt% Cr_2_O_3_ in propanetriol monodecanoate dioctanoate (Mytrol 318, BASF S.r.l, Monheim, Germany) and then cleaned according to the cleaning methods reported in ISO 10545-14 [44].

The scratch resistance (SR) was carried out as reported in ASTM C1895-20, using metallic picks as indenters [45]. The instrument ensures a constant load of 31.4 ± 1.9 N at the contact point between the pick and the sample, and a slope of 70° concerning the sample surface. Scratches were made by sliding the picks along the surface of one 15 × 15 cm sample cut from 30 × 30 cm ceramic tiles.

ISO/CD 10545-22 [37] also provides a classification in three different classes as far as floor ceramic tiles are considered, and the requirements for each class are reported in Table 4. The three classes, named H, HH, and HHH, are connected to the most popular intended use for ceramic tiles as follows: samples classified in H class are suitable for residential environments (e.g., bedrooms, living rooms, etc.), samples classified in HH are suitable for application in low-traffic areas (e.g., restaurants, offices, etc.), and samples classified in HHH are suitable for high-traffic area (e.g., industrial environments, loading and unloading areas, etc.). Based on the outcomes of the performed tests, it has been possible to classify the investigated glazed ceramic tiles.

All the tests were performed at room temperature (25 ± 3 °C) and a relative humidity of 45% ± 5%.

## 4. Results and Discussion

**Figure 2:**
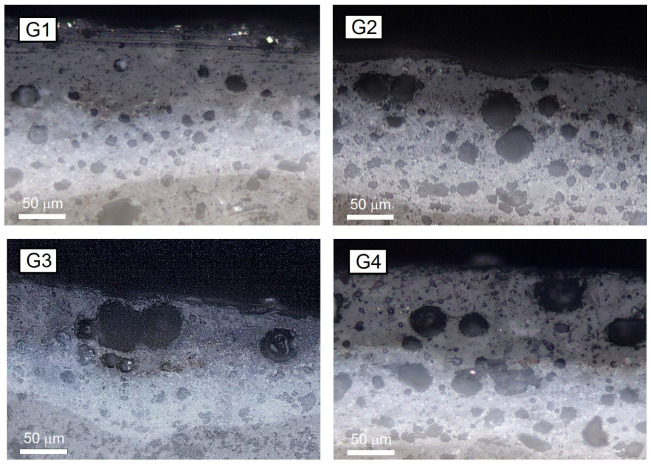
Stratigraphic analysis of G1, G2, G3, and G4 glazed tiles.

The designed GCP was validated by testing G1, G2, G3, and G4 glazes. Optical observations were carried out on polished cross-sections of the four investigated glazes to measure the thicknesses of both the engobe and glaze layers. The analyzed cross-sections are reported in Figure 2. The airless application of engobe and glaze layers on the body surface results in an uneven coating, leading to notable variations in the thickness of each layer after the firing process. The outcome of the image analyses highlights that engobe thickness is in the range between 40 and 70 µm, while glaze thickness ranges from 30 to 60 µm. Comparing all the samples, no significant variations were observed between the glazes, due to the fact that the same amount (44 mg/cm^2^) of engobe and glaze slurries was deposited on the green body surface. Micrographs for G1 reveal a small number of closed pores with diameters ranging from 10 to 20 µm. Conversely, a high number of closed pores are observed in G2, G3, and G4, exhibiting larger diameters in the range between 30 and 50 µm. Additionally, a large number of small closed porosities ranging from 5 to 30 µm are also observed in the engobe layer. Closed pores can be primarily attributed to the gas entrapment caused by substrate degasification during firing [46,47]. The entrapment of gas bubbles within the glaze layer also depends on its viscosity and fusibility in correspondence to the peak temperature of the industrial firing cycle (T_P_ = 1205 °C) [48].

**Figure 3:**
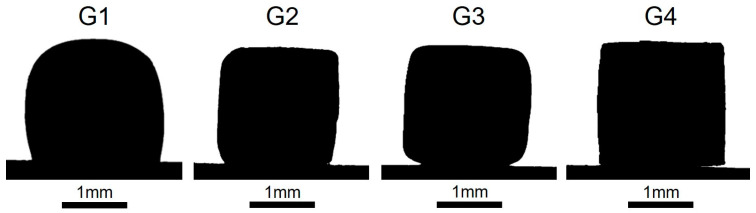
Shape of the G1, G2, G3 and G4 glaze cylindrical pellets at T_P_ by hot stage microscope analysis. The glaze cylindrical pellet dimensions before the HSM analysis correspond to a diameter of 2 mm and a height of 3 mm.

For this reason, glaze fusibility was assessed by determining their characteristic temperatures (Table 5), whereas viscosity was qualitatively evaluated by analyzing the shape of the cylindrical pellets in correspondence of T_P_ during the hot stage microscope analysis (Figure 3) [47,48,49,50].

In correspondence to T_P_ = 1205 °C, G1 falls within the range defined by sphere (T_SP_ = 1203 °C) and hemisphere temperatures (T_HSP_ = 1220 °C) (T_SP_ < T_P_ < T_HSP_). The shape of the G1 glaze pellet suggests that at T_P_, G1 is losing its structural stiffness, indicating a reduction in its viscosity. Conversely, G2 and G3 result in the softening stage (T_SO_ < T_P_ < T_SP_), with T_SO_ = 1180 °C and T_SP_ = 1232 °C for G2 and T_SO_ = 1170 °C and T_SP_ = 1210 °C for G3. These two glazes exhibit a rounding of the corners due to the coexistence of liquid and solid states. Finally, G4 results in the sintering phase (T_SI_ < T_P_ < T_SO_) with T_SI_ = 1197 °C and T_SO_ = 1240 °C. At T_P_, G4 exhibits shrinkage without any change in shape, suggesting the highest viscosity among the four samples.

Sample G1, exhibiting the highest fusibility, results in the least viscous glaze and, accordingly, demonstrates a reduced tendency to form closed pores. Sample G4 exhibits refractory behavior, characterized by low fusibility and high viscosity. Furthermore, it can be observed that the higher the T_SO_ value of the glaze, the higher its viscosity at T_P_, and this can promote gas entrapment with the formation of closed pores with different dimensions [47].

**Figure 4:**
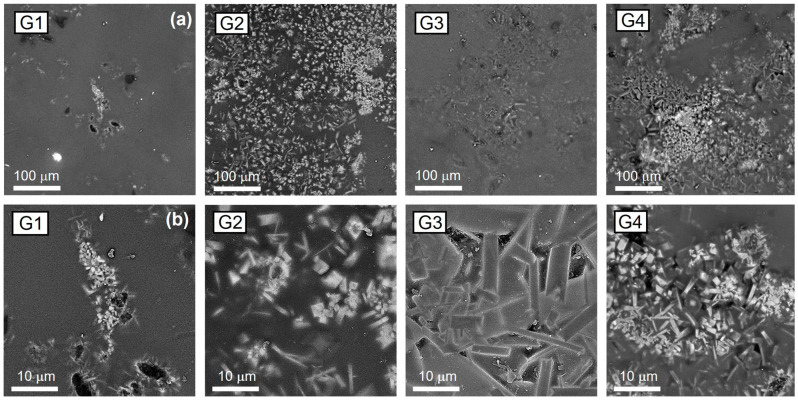
Low- (**a**) and high-magnification (**b**) SEM observations of the surface of G1, G2, G3, and G4 acquired backscattered electrons.

The microstructural analysis was carried out by SEM on the four sample surfaces (Figure 4) by acquiring backscattered electrons.

Low-magnification micrographs reveal the distribution of crystalline phases within the amorphous matrix, while high-magnification micrographs provide information about crystal shape and size. G1, G2, and G4 exhibit a microstructure composed of square-shaped and bar-shaped crystals, whereas G3 exhibits exclusively bar-shaped ones. Furthermore, G1 exhibits the smallest crystals (<5 μm) among the observed glazes, mainly randomly dispersed in a few crystalline clusters. In contrast, G2, G3, and G4 display crystals homogeneously dispersed within the glassy phase, with the largest crystals (>20 μm) clearly evident in G3. Comparing all the results already presented (microstructural observations and thermal and optical analyses), it was found that G1 exhibits the highest fusibility, the lowest viscosity at T_P_, closed pores with diameters ranging from 10 to 20 µm, and the lowest crystallinity. Conversely, all the other glazes show higher crystallinity and increases in T_SI_, T_SO_, and T_SP_ moving from G2 to G4. This increase promotes a rise in viscosity at T_P_, which, in turn, generates a larger number of closed pores with dimensions ranging from 30 to 50 µm. The relationship found between viscosity and crystallinity confirms what is already reported in the literature [51].

**Figure 5:**
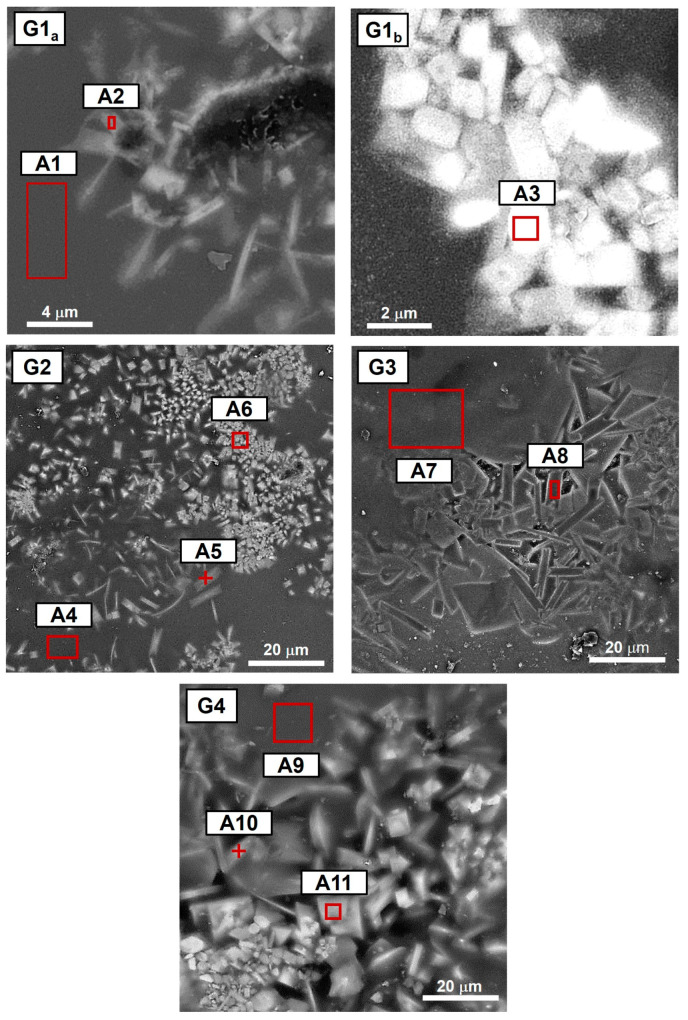
SEM observations of the surfaces of G1, G2, G3, and G4 glaze with areas (A1 to A11) where EDS analysis was carried out. G1_a_ and G1_b_ refer to two different areas of G1.

**Figure 6:**
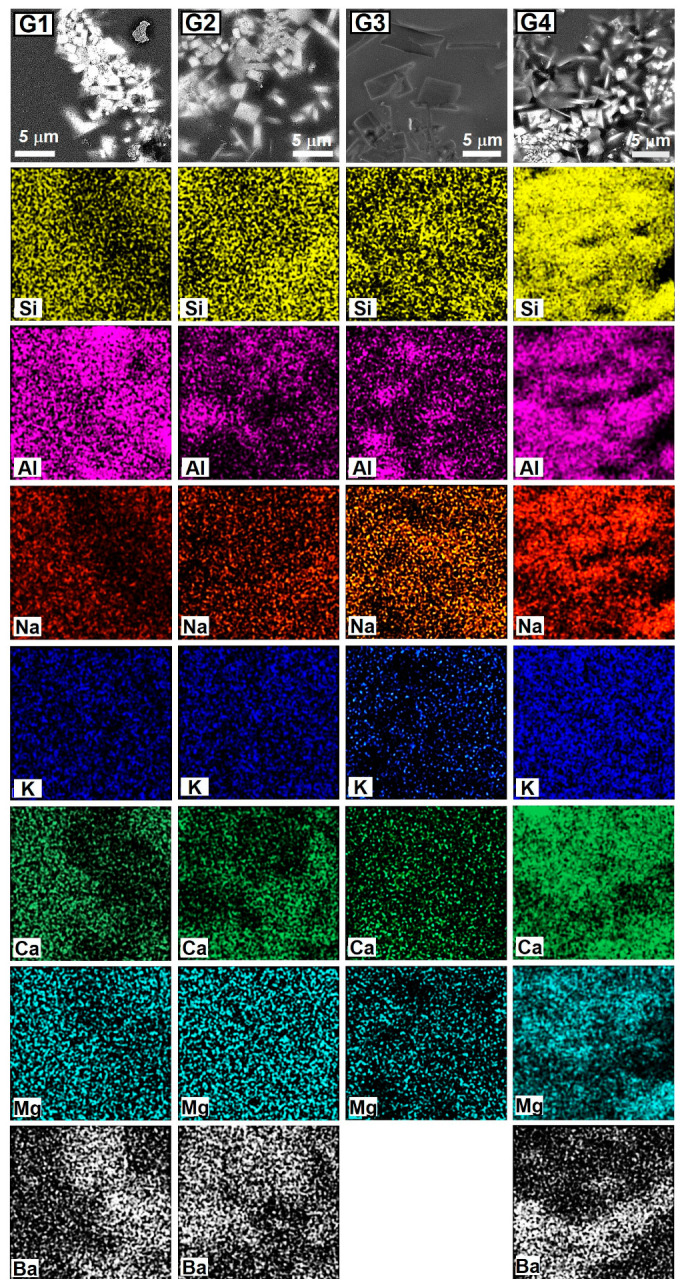
Qualitative elemental maps obtained investigating the free surfaces of G1, G2, G3, and G4.

The chemical composition of both crystalline and amorphous phases was determined through EDS analysis. The considered areas are shown in Figure 5, while Table 6 reports the elemental compositions. Additionally, Figure 6 displays X-ray maps providing qualitative information about element distribution in amorphous and crystalline phases. G1, G2, and G4 exhibit a higher concentration of Ba in the crystalline phases compared to that measured in the amorphous matrix. Furthermore, X-ray maps indicate an increased presence of Ba and Al and a decreased concentration of Na, Ca, and Si in correspondence to crystalline phases. Conversely, in G3, both EDS and X-ray maps are unable to provide significant differences between the elemental composition of crystalline and amorphous phases, except for a higher concentration of Al found in the former.

**Figure 7:**
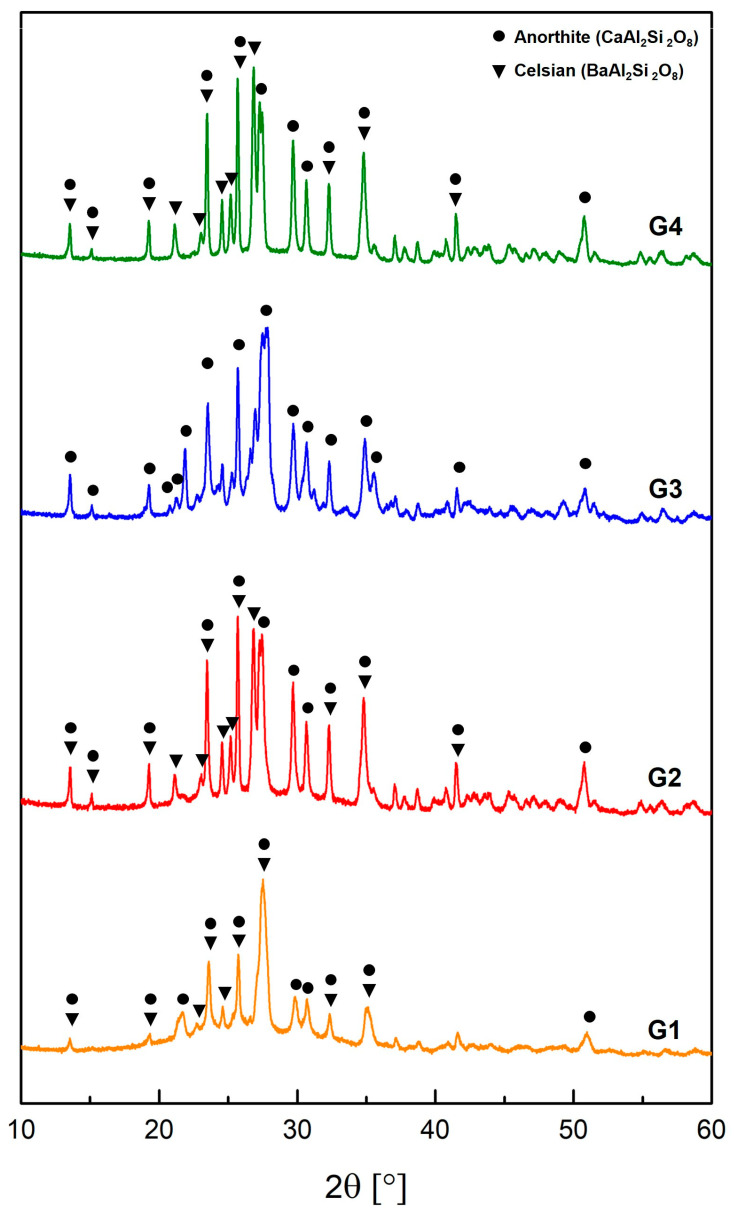
XRD diffraction patterns of G1, G2, G3, and G4.

The results of SEM investigations have been also compared with micrographs and crystalline habits reported in the literature [11,14,16,17,19,21,27,52,53,54]. This comparative analysis highlights that the square-shaped crystals containing Ba, observed in G1, G2, and G4, may correspond to celsian (BaAl_2_Si_2_O_8_) [16,27,52,53,54]. Conversely, the bar-shaped crystals, clearly visible in all the samples (and especially in G3), may correspond to anorthite (CaAl_2_Si_2_O_8_) [5,11,14,19]. To further support these associations, XRD analysis was performed on G1, G2, G3, and G4 samples, and the results are presented in Figure 7.

As expected, XRD diffraction patterns of G1, G2, and G4 show the presence of both celsian (BaAl_2_Si_2_O_8_, ICSD 98-028-1283) and anorthite (CaAl_2_Si_2_O_8_, ICSD 98-012-0301), whereas XRD diffraction pattern of G3 exclusively reveals the presence of anorthite. In addition, a broad hump is observed in the 2θ range between 20° and 30° in G1 pattern. This hump is the result of the X-ray scattering due to the absence of long-range atomic order in the structure [55], thus suggesting that G1 displays a reduced crystallization when compared to G2, G3, and G4 [56,57].

Celsian crystallization is surely influenced by BaO content, as well as by other oxides, such as TiO_2_, which, when added to glass-ceramic, acts as a nucleating agent [58,59]. Therefore, although in G1 the BaO content is less than 10 wt%, the crystallization of BaAl_2_Si_2_O_8_ still occurs, probably fostered by the presence of TiO_2_ in trace amounts (<1 wt%). For G2 and G4, the higher quantity of BaO and the presence of CaO justify the coexistence of both CaAl_2_Si_2_O_8_ and BaAl_2_Si_2_O_8_ [14,16,19,27,32]. For G3, the higher CaO content (8–12 wt%), coupled with low MgO presence (1–3 wt%), induces the crystallization of anorthite [11]. Moreover, it is known that the presence of B_2_O_3_ (present in G3 in the range of 0.5–1%) in glaze composition enhances both the quantity and size of the anorthite crystal phase, reducing light scattering and improving transparency [11,14].

**Figure 8:**
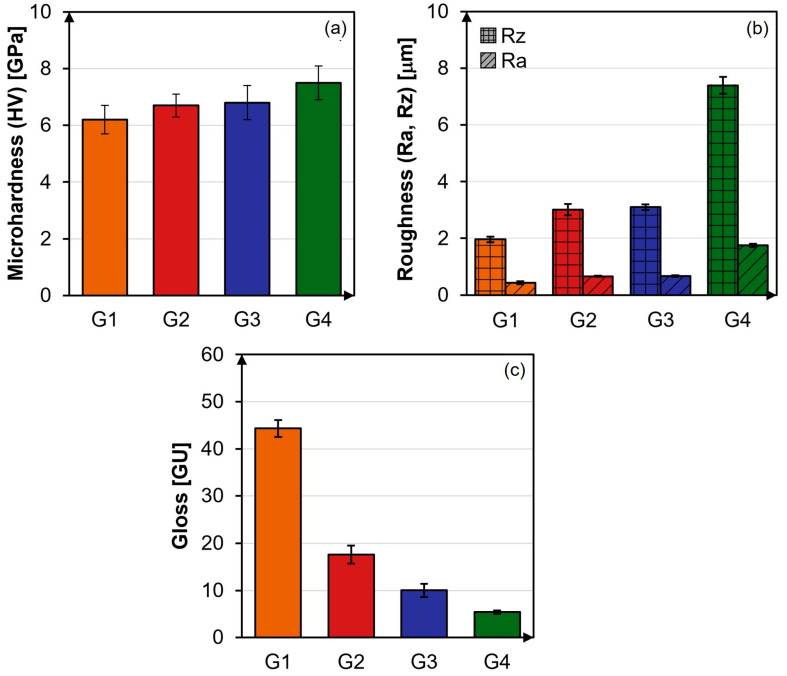
Results of: (**a**) HV test, (**b**) Rz and Ra roughness analysis, and (**c**) gloss measurements carried out on G1, G2, G3, and G4.

To understand how microstructure influences the surface performance of glazed tiles, results of Vickers microhardness, average roughness, and the maximum height of the roughness profile, as well as gloss measurements, are reported in Figure 8.

Vickers microhardness (HV) is an important parameter to be considered in GCP since it defines the material ability to resist permanent deformations. The HV values for G1, G2, and G3 are all lower than 7 GPa, as reported in Figure 8a. Specifically, G1 reveals the lowest HV value (6.2 ± 0.5 GPa), G2 and G3 exhibit comparable values (6.7 ± 0.4 GPa and 6.8 ± 0.6 GPa, respectively), and G4 exhibits the highest (7.5 ± 0.6 GPa), making it the hardest one. The low HV value obtained for G1 agrees with the few crystalline clusters observed by SEM micrographs reported in Figure 4. Typically, the glassy phase exhibits HV values lower than those of the crystalline phases [9,14]. Comparing the obtained results with literature data, the HV value obtained for G1 agrees with that of amorphous glazes (HV ≈ 5.9 GPa) [9,14], whereas G2, G3, and G4 display HV values comparable to those measured for glass-ceramic glazes [9,11,14,57]. Finally, the HV value of G3, whose microstructure is exclusively composed of CaAl_2_Si_2_O_8_, is comparable to those measured by Wang et al. on anorthite-based glass-ceramic glazes [14].

The evaluation of roughness parameters provides specific information on glazed ceramic tile surface texture. Roughness parameters are particularly important as they affect ceramic tile suitability for specific intended uses. Indeed, surface texture significantly influences ceramic tile slip resistance, making its assessment crucial to ensure pedestrian safety and prevent slip-and-fall injuries. Therefore, roughness parameters such as average roughness (Ra) and the maximum height of the roughness profile (Rz) have also been determined for G1, G2, G3, and G4 and reported in Figure 8b. As Ra only partially describes surface texture [11,14,15,30], Rz has also been considered. The lowest and highest Ra and Rz values have been found for G1 and G4 samples, resulting in the smoothest and roughest surfaces, respectively. Furthermore, Ra and Rz values measured for G2 and G3 are comparable. The trend in roughness parameters reflects the one found for Vickers microhardness, as both characteristics are closely related to crystalline phases [60].

Another important property of glaze coatings is the surface gloss. Concerning gloss evaluation (Figure 8c), G1 and G4 exhibit the highest and the lowest gloss values, respectively, whereas those of G2 and G3 are comparable. Gloss values are also influenced by crystallinity, as the quantity, shape, and size of crystalline phases also affect optical properties, which in turn impact the aesthetic appearance of ceramic tiles. Amorphous glaze typically displays a refractive index comparable to that of glass (i = 1.51), while the refractive index of crystalline phases is generally higher [14,61], especially when high molecular weight atoms (e.g., Ba) are present in their lattice [53].

The assessment of glaze tribomechanical performances in the ceramic tile manufacturing industry is commonly performed following test methods reported in ISO standards. For this reason, the application of GCP has involved the adoption of the following tests: impact resistance (ISO 10545-5 [35]), surface abrasion resistance (ISO 10545-7 [36]), and wear resistance (ISO/CD 10545-22 [37]). The outcomes of the impact resistance test reveal the same restitution coefficient (e) for each of the tested samples (e_G1_ = 0.88, e_G2_ = 0.88, e_G3_ = 0.89, e_G4_ = 0.88). Additionally, after the visual inspection, no fractures, craters, or chipping were observed on ceramic tile surfaces, thus indicating that this test does not discriminate the performances of the glazes when their Vickers microhardness falls between 6.2 and 7.5 GPa.

**Figure 9:**
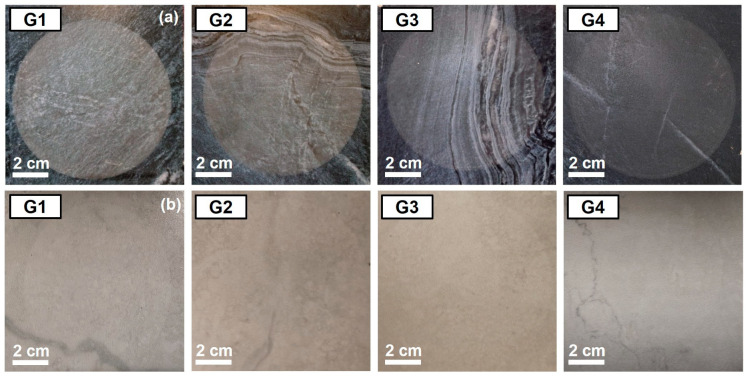
Surface abrasion damages on (**a**) dark blue decorated samples (G1 to G4) after 600 abrasion cycles and (**b**) light gray decorated samples (G1 to G4) after 1200 abrasion cycles. The test was carried out according to ISO 10545-7.

The surface abrasion resistance test was carried out according to ISO 10545-7 for each glaze formulation on light gray and dark blue decorated tiles. The abraded areas of the tested tiles are reported in Figure 9. For dark blue samples, all the four glazes (G1, G2, G3, and G4) reveal an abraded area visible after 600 abrasion cycles, thus resulting in class 2 according to the classification system reported in ISO 10545-7. Class 2 refers to “floor tiles suitable for moderate foot traffic areas with small amounts of abrasive dirt (e.g., private residential rooms)” according to Annex-N of ISO 13006 [22]. Conversely, for light gray samples, none of the four glazes show any visible damage even after 12000 abrasion cycles, thus resulting in class 5 classification, which refers to “floor tiles suitable for long-term heavy pedestrian traffic (e.g., shopping mall, airports, hospitals, and others)” [22]. Thus, for this test, the color of the decoration plays a very important role in the final classification regardless of the chemical composition/microstructure of the investigated glazes [12].

The results obtained by applying the wear resistance method according to ISO/CD 10545-22 [37] and relevant classification are reported in Table 7. It was observed that all tested samples exhibited a ΔWL_S_ lower than 0.028 mg/cm^2^, initially resulting in class HHH. This was somehow expected, as these glazes are designed for high firing temperatures (about 1205 °C), thus suitable for porcelain stoneware tiles. The assessment of scratch resistance revealed that G1, G2, and G3 have the same value (6), thus achieving class HH, while G4 reaches class HHH, as its scratch resistance value is equal to 7.

The final classification is the result of the lowest performance of the different methods carried out. The assessment of ΔG highlights that G1 exhibits the highest value, resulting in class H; G2 and G3 are comparable, resulting in class HH; and G4 exhibits the lowest ΔG value, resulting in class HHH. Stain resistance was evaluated by applying green chromium stains on the abraded area and removing them according to the procedure reported in ISO 10545-14 [44]. Stains were effectively removed by washing with water, thus resulting in class 5 classification, the highest class according to the ISO 10545-14. Thus, summarizing the results, ΔWL_S_ was not able to discriminate between the investigated samples, whereas both scratch resistance and ΔG give different results as a function of the different glazes. In particular, ΔG was found to be the most critical parameter for the final classification, as it is based on the results of the lowest performance of the different methods carried out. Accordingly, G4 and G1 have been classified as HHH and H, respectively, whereas G3 and G2 belong to the HH class. Relating these outcomes with intended use classifications, it is determined that G1 is suitable for residential environments, G2 and G3 for low-traffic areas, and G4 for high-traffic ones.

**Figure 10:**
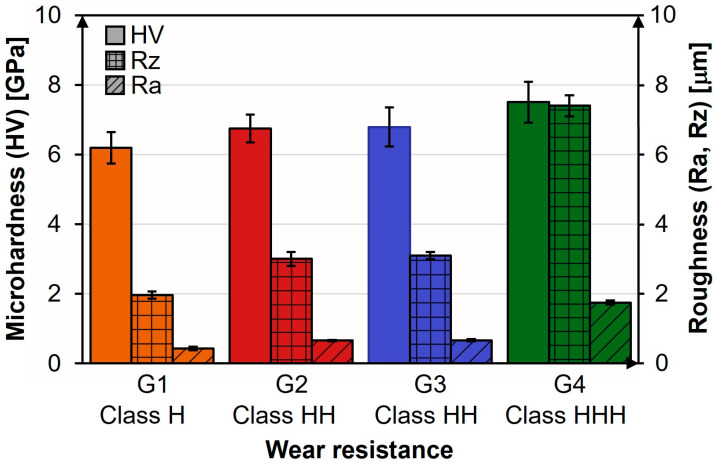
Correlation between Vickers microhardness (HV), average roughness and maximum height of the roughness profile (Ra and Rz) parameters, and wear resistance class.

The wear resistance test method can distinguish between the investigated glazes much more effectively than the surface abrasion test method described in ISO 10545-7. Moreover, comparing the results of the analytical test with those obtained by the wear resistance method, a qualitative relationship is found, as reported in Figure 10.

G1, exhibiting the lowest HV, Ra, and Rz values and the highest initial gloss according to the lowest content of crystalline phases, falls into the H class. Conversely, G4, characterized by the highest HV, Ra, and Rz values and the lowest initial gloss according to its high crystallinity, falls into the HHH class.

## 5. Conclusions

This study aimed to design and validate a glaze characterization protocol that can be used by glaze manufacturers and researchers involved in new glaze formulations. The protocol was applied to four different glazes, and the main results can be interpreted as follows:

⮚ Analytical tests provide fundamental information that is strongly related with the chemical composition of the glaze and the crystalline phases formed during firing. A complete microstructural analysis involving the techniques listed in the protocol allows for the determination of characteristics that are strongly connected to the nature, shape, and distribution of the crystalline phases;
⮚ Technological tests give different results, as some of them (e.g., impact resistance and surface abrasion resistance tests) are not able to differentiate between the investigated glazes, whereas others, such as the combination of tests present in the wear resistance test, seem very effective in highlighting the difference between the various surface properties of the glazes;
⮚ A good correlation between the analytical tests and wear resistance test has been found, highlighting that for ceramic tile glaze formulation purposes, all these tests can be largely adopted.

The application of the characterization protocol to the investigated glaze provides a set of target parameters (HV, Ra, Rz, G, ΔWL_S_, ΔG (@ 600), ΔG (@ 6000), scratch resistance), each with a specific threshold value. Among these, HV > 7.5 GPa, ΔG (@ 6000) < 5, and scratch resistance > 7 are considered the most relevant, to be used as a starting point for the development of glass-ceramic glaze formulations with high tribomechanical performance and good aesthetic appeal over time. Future developments will involve the adoption of this new glaze characterization protocol to develop enhanced glass-ceramic glazes for porcelain stoneware tiles for high traffic flooring applications. This protocol will be promoted in both glaze and ceramic tile manufacturing sectors with the goal to develop an ISO technical report for further strengthening the tribo-mechanical performances of ceramic tiles.

## Figures and Tables

**Table 1:** Formulation of the four investigated glazes (wt%).

Frits/Raw Materials	G1	G2	G3	G4
F1	30–40	-	30–40	10–20
F2	-	20–30	-	10–20
F3	10–20	20–30	-	
F4	-	1–5	5–10	
Clay	5–10	5–10	-	5–10
Kaolin	10–20	5–10	5–10	-
Dolomite	5–10	-	1–5	5–10
Feldspar	10–20	10–20	5–10	10–20
Nepheline	-	10–20	10–20	10–20
Quartz	10–20	-	5–10	1–5
BaCO_3_	5–10	1–5	-	5–10
ZnO	1–5	1–5	-	1–5

**Table 2:** Oxide composition (range in wt%) of both frits and raw materials. L.O.I. stands for loss of ignition.

Frit/Raw Material	SiO_2_	Al_2_O_3_	MgO	CaO	Na_2_O	K_2_O	BaO	B_2_O_3_	ZnO	TiO_2_	L.O.I.
F1	50–60	10–20	-	10–20	-	1–5	-	-	5–10	-	-
F2	30–40	10–20	-	20–30	-	1–5	30–40	-	-	-	-
F3	40–50	10–20	-	20–30	1–5	-	-	-	-	-	-
F4	30–40	10–20	-	10–20	-	1–5	-	1–5	-	-	-
Clay	58	29	0.3	0.3	0.2	1.1	-	-	-	0.7	10.5
Kaolin	47	38.5	0.2	0.1	-	1.6	-	-	-	0.2	12.4
Dolomite	-	-	21.6	31.8	-	-	-	-	-	-	47.6
Feldspar	70	18	0.3	0.3	10.5	0.5	-	-	-	-	0.4
Nepheline	60.8	23.4	-	0.4	9.8	5	-	-	-	-	0.6
Quartz	99.3	0.4	-	0.05	-	0.05	-	-	-	-	0.2
BaCO_3_	-	-	-	-	-	-	76.5	-	-	-	23.5

**Table 3:** Oxide composition (range in wt%) of the investigated glazes. L.O.I stands for loss of ignition.

Samples	SiO_2_	Al_2_O_3_	Na_2_O	K_2_O	MgO	CaO	BaO	ZnO	B_2_O_3_	TiO_2_	L.O.I
G1	50–55	16–18	3–5	1–3	1–3	6–10	6–8	1–3	-	<1	5–7
G2	45–50	12–16	3–5	1–3	1–3	5–8	15–20	1–3	-	<1	1–3
G3	50–55	17–20	3–5	1–3	1–3	8–12	-	3–5	0.5–1	<1	2–4
G4	45–50	18–21	3–5	1–3	1–3	4–7	9–12	3–5	-	<1	5–7

**Table 4:** Wear resistance test classification according to ISO/CD 10545-22 (ΔWL_S_: specific weight loss; ΔG: gloss variation) [37].

Test Method	Wear Resistance Class		
	H	HH	HHH
ΔWL_S_ [mg/cm^2^] (@ 6000)	0.035 < ΔWL_S_ < 0.045	0.028 ≤ ΔWL_S_ < 0.035	ΔWL_S_ < 0.028
ΔG [GU]	@ 600 cycles 36 ≤ ΔG ≤ 50	@ 600 cycles 6 < ΔG ≤ 35	@ 6000 cycles ΔG ≤ 5
Stain resistance	Minimum class: 3		
SR	Hardness point ≥ 4	Hardness point ≥ 5	Hardness point ≥ 7

**Table 5:** Characteristic temperatures of G1, G2, G3, and G4. T_SI_, T_SO_, and T_SP_ stand for sintering, softening, and hemisphere temperatures, respectively.

Samples	T_SI_	T_SO_	T_SP_
	[°C]	[°C]	[°C]
G1	1138	1160	1203
G2	1150	1180	1232
G3	1146	1170	1210
G4	1197	1240	1256

**Table 6:** EDS data expressed in atomic percentage (at%) obtained by analyzing the areas reported in Figure 5 for G1, G2, G3, and G4.

Sample	Area	O	Na	K	Ca	Mg	Zn	Ba	Al	Si
G1	A1	58.3 ± 2.3	5.9 ± 0.4	1.1 ± 0.2	5.7 ± 0.6	1.8 ± 0.1	1.0 ± 0.1	1.3 ± 0.2	10.4 ± 1.3	10.4 ± 0.8
	A2	59.3 ± 2.3	4.0 ± 0.7	0.7 ± 0.2	3.4 ± 1.2	0.6 ± 0.2	0.3 ± 0.1	2.6 ± 1.2	14.6 ± 2.3	14.6 ± 2.3
	A3	58.4 ± 2.8	2.9 ± 0.7	0.7 ± 0.2	2.2 ± 0.6	0.4 ± 0.1	0.3 ± 0.1	4.0 ± 1.3	12.0 ± 1.2	19.1 ± 1.7
G2	A4	55.7 ± 2.8	4.6 ± 0.1	0.6 ± 0.1	7.5 ± 0.3	0.3 ± 0.1	1.2 ± 0.1	2.1 ± 0.1	7.1 ± 0.6	20.9 ± 1.1
	A5	62.6 ± 1.3	3.5 ± 0.2	0.7 ± 0.1	6.0 ± 1.4	0.2 ± 0.1	1.0 ± 0.3	2.7 ± 0.2	7.0 ± 1.8	16.5 ± 1.2
	A6	59.1 ± 1.2	3.0 ± 0.2	1.0 ± 0.1	3.7 ± 0.7	0.1 ± 0.1	0.7 ± 0.1	4.4 ± 0.4	10.2 ± 0.5	17.9 ± 1.1
G3	A7	58.4 ± 1.1	4.5 ± 0.2	1.4 ± 0.5	5.7 ± 0.6	1.4 ± 0.5	1.6 ± 0.2	-	6.7 ± 0.4	20.3 ± 0.8
	A8	56.9 ± 1.2	4.2 ± 0.5	0.4 ± 0.1	6.5 ± 0.6	0.9 ± 0.2	0.7 ± 0.2	-	11.2 ± 1.1	19.3 ± 1.1
G4	A9	43.7 ± 2.2	5.0 ± 0.3	1.3 ± 0.2	4.4 ± 0.9	2.6 ± 0.7	3.2 ± 0.8	3.7 ± 1.2	9.6 ± 0.2	26.5 ± 1.2
	A10	40.6 ± 3.4	5.0 ± 0.2	0.8 ± 0.1	4.5 ± 0.8	2.6 ± 0.7	2.9 ± 1.2	6.0 ± 1.1	11.4 ± 0.3	26.1 ± 1.3
	A11	49.0 ± 2.1	3.9 ± 0.3	0.9 ± 0.2	3.6 ± 1.1	1.5 ± 0.7	1.9 ± 1.1	9.2 ± 2.5	9.6 ± 0.5	20.5 ± 2.6

**Table 7:** Results of wear resistance performed according to ISO/CD 10545-22 and relevant classification.

Sample	G1	G2	G3	G4
ΔWL_S_ [g/cm^2^]	0.025 ± 0.001	0.018 ± 0.001	0.025 ± 0.001	0.017 ± 0.001
ΔG (@ 600) [GU]	38.7 ± 0.9	12.8 ± 0.8	14.1 ± 1.2	-
ΔG (@ 6000) [GU]	-	-	-	2.5 ± 1.3
Stain resistance	5	5	5	5
Scratch resistance	6	6	6	7
Wear resistance class	H	HH	HH	HHH

## Data Availability

The data presented in this study are available on request from the corresponding author. The data are not publicly available due to confidentiality reasons.
